# CaSR-Mediated hBMSCs Activity Modulation: Additional Coupling Mechanism in Bone Remodeling Compartment

**DOI:** 10.3390/ijms22010325

**Published:** 2020-12-30

**Authors:** Hyunji Cho, Jisoo Lee, Seoyoung Jang, Jungsun Lee, Tong In Oh, Youngsook Son, EunAh Lee

**Affiliations:** 1 College of Life Science and Graduate School of Biotechnology, Kyung Hee University, Seochon-dong, Kiheung-go, Yongin-si, Geonggi-do 17104, Korea; hj0522a@hanmail.net; 2 Department of Medical Engineering, Graduate School, Kyung Hee University, Seoul 02447, Korea; leex5812@khu.ac.kr (J.L.); syjang329@khu.ac.kr (S.J.); 3 R&D Institute, Biosolution Inc., Seoul 18111, Korea; dvmljs@biosolutions.co.kr; 4 Department of Biomedical Engineering, School of Medicine, Kyung Hee University, Seoul 02447, Korea; tioh@khu.ac.kr; 5 Department of Genetic Engineering, College of Life Science, Kyung Hee University, Seochon-dong, Kiheung-go, Yongin-si, Geonggi-do 17104, Korea; 6 Impedance Imaging Research Center, Kyung Hee University, Seoul 02447, Korea

**Keywords:** bone remodeling, calcium-sensing receptor, proliferation, osteoblast-osteoclast coupling

## Abstract

Near the bone remodeling compartments (BRC), extracellular calcium concentration (Ca^2+^_o_) is locally elevated and bone marrow stromal cells (BMSCs) close to the BRC can be exposed to high calcium concentration. The calcium-sensing receptor (CaSR) is known to play a key role in maintaining extracellular calcium homeostasis by sensing fluctuations in the levels of extracellular calcium (Ca^2+^_o_). When human BMSCs (hBMSCs) were exposed to various calcium concentrations (1.8, 3, 5, 10, 30 mM), moderate-high extracellular calcium concentrations (3–5 mM) stimulated proliferation, while a high calcium concentration (30 mM) inhibited the proliferation. Exposure to various calcium concentrations did not induce significant differences in the apoptotic cell fraction. Evaluation of multi-lineage differentiation potential showed no significant difference among various calcium concentration groups, except for the high calcium concentration (30 mM) treated group, which resulted in increased calcification after in vitro osteogenic differentiation. Treatment of NPS2143, a CaSR inhibitor, abolished the stimulatory effect on hBMSCs proliferation and migration indicating that CaSR is involved. These results suggest that the calcium concentration gradient near the BRC may play an important role in bone remodeling by acting as an osteoblast–osteoclast coupling mechanism through CaSR.

## 1. Introduction

Calcium plays an essential role in all living organisms, accounting for about 1–2% of adult human body weight. About 99% of total body calcium is found in the mineral phase of bone and teeth, and the remaining 1% is found in the blood, extracellular fluid, and soft tissues. Among these, extracellular calcium (Ca^2+^_o_) concentration is constantly maintained within the range of 1.1–1.3 mM [1] and resting level of intracellular calcium (Ca^2+^_i_) concentration is tightly regulated as low as about 100 nM [2]. As an increase in Ca^2+^_i_ concentration plays an important role as an intracellular signaling component, it is important to sense Ca^2+^_o_ and maintain a constant level of Ca^2+^_o_ concentration [2].

Calcium-sensing receptor (CaSR) plays a key role in Ca^2+^_o_ homeostasis by sensing small fluctuations as low as 200 μM of Ca^2+^_o_ and modulating various signaling pathways [3].

CaSR, a G-protein coupled receptor, has three structural domains including large amino-terminal extracellular domain (ECD) containing Ca^2+^_o_ binding sites, seven transmembrane domains (TMD), and a carboxyl-terminal tail. The ECD of CaSR contains Cys-rich region and participates in disulfide bonds for CaSR dimerization [4]. Binding of Ca^2+^_o_ to Ca^2+^_o_ binding sites induces conformational change on CaSR, and activates coupled G-proteins. Activation of CaSR modulates numerous signaling pathways including the PKC signaling pathway mediated by G_q/11_, the MAPK signaling pathway mediated by G_12/13_, and the inhibition of adenylase cyclase mediated by G_i_, regulating various cellular processes [4]. CaSR is expressed in several tissues such as parathyroid, intestine, bone, and cartilage [5,6,7,8] and in a variety of cell types such as parathyroid cells, myeloma cells, fibroblasts, osteoblasts, and osteoclasts [9,10,11,12,13] to play a critical role in tissues maintaining Ca^2+^_o_ homeostasis.

In the bone, bone remodeling involving osteoclastic bone resorption and osteoblastic bone formation occurs at discrete sites named as bone remodeling compartments (BRCs) [14]. During bone resorption, deposited calcium is released from mineralized bone matrix through the action of bone resorption by osteoclasts and this constitutes the Ca^2+^_o_ gradient present around the BRC. It is reported that Ca^2+^_o_ near resorbing osteoclasts is elevated as high as 40 mM [15]. Previous studies have reported that the Ca^2+^_o_ gradient has several effects on the cells residing in BRCs including osteoblasts and osteoclasts. High Ca^2+^_o_ level stimulates proliferation and chemotaxis of osteoblasts [12,16] and induces differentiation of precursor cells into mature osteoblasts, thereby facilitating mineralization [17,18]. In addition, a high Ca^2+^_o_ level inhibits osteoclastic activity and stimulates the apoptosis of osteoclasts [13].

CaSR is expressed in bone marrow stromal cells (BMSCs) [19] as well as osteoblast-like cells and osteoclast-like cells in humans and mice [8,13,16]. It is expected that a localized high calcium level produces the signal to inhibit bone resorption and stimulates bone formation by modulating the activity of hBMSCs toward bone formation.

In this study, to examine the response of hBMSCs to high Ca^2+^_o_, the effect of high Ca^2+^_o_ on hBMSCs was investigated from various aspects including cell proliferation, survival, differentiation, and chemotaxis. Human dermal fibroblasts (hDFs) were compared as a control because hDFs reside in a microenvironment of constant Ca^2+^_o_ level. The results showed that an increase of Ca^2+^_o_ stimulates hBMSC proliferation and migration in a dose-dependent manner in a CaSR dependent way, based on the fact that NPS2143, a selective CaSR antagonist, abolishes the effects caused by a high Ca^2+^_o_ concentration [20].

## 2. Results

### 2.1. Increased Proliferation of Human Bone Marrow Stromal Cells (hBMSCs) in Moderate-High Calcium Concentration Is Mediated by Calcium-Sensing Receptor (CaSR)

**Figure 1:**
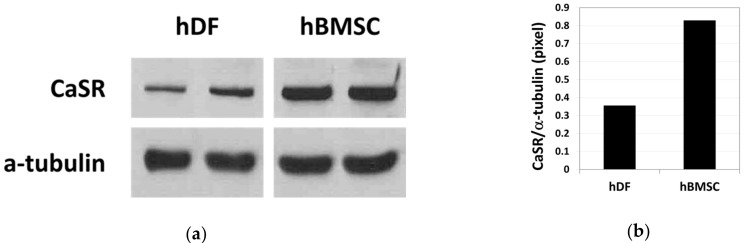
Basal level expression of calcium-sensing receptor (CaSR) in human dermal fibroblasts (hDFs) and human bone marrow stromal cells (hBMSCs) examined by western blot analysis (**a**) and the relative density of CaSR band normalized to α-tubulin (**b**) (*n* = 2).

The basal expression level of CaSR (a 140 kDa protein) in hDFs and hBMSCs was compared by western blot analysis (Figure 1a, Supplementary Figures S1 and S2). Relative band intensity indicates that the basal level expression of CaSR was 2.3-fold higher in hBMSCs compared with hDFs (Figure 1b).

**Figure 2:**
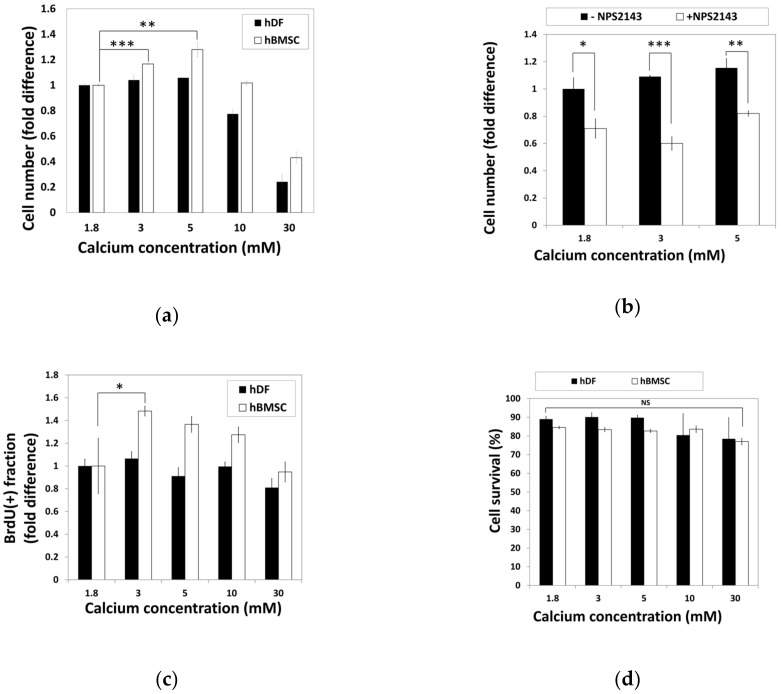
Moderate-high calcium concentration stimulate hBMSC proliferation through CaSR. (**a**) hDFs and hBMSCs were treated with various calcium concentrations for 48 h and relative cell numbers were analyzed by the MTT assay (** *p* = 0.0038, *** *p* = 0.0002). (**b**) hBMSCs were treated with NPS2143, a CaSR antagonist. Relative cell density was determined by the MTT assay (* *p* = 0.0176, ** *p* = 0.0034, ** *p* = 0.0004). (**c**) During hBMSC exposure to various calcium concentrations for 48 h, BrdU was treated for the last 24 h. Proportion of BrdU-positive cells was compared as fold difference (* *p* = 0.0416). (**d**) Comparison of cell survival in response to various calcium concentrations based on the propidium iodide-incorporated apoptotic assay. hDFs and hBMSCs were exposed to various calcium concentrations for 48 h and subjected to propidium iodide (PI) staining. The ratio of PI-negative survival fractions in each group of cells was analyzed by fluorescence-activated cell sorting (FACS) and compared.

To examine the effect of calcium concentration on cell activity, hBMSCs and hDFs were cultured in standard media containing various calcium concentrations for 48 h before relative cell numbers were analyzed by the 3-(4,5-dimethylthiazol-2-yl)-2,5-diphenyltetrazolium bromide (MTT) assay (Figure 2a). The cell number of hBMSCs increased in the media containing moderate-high calcium concentrations (3–5 mM), while the hDFs showed no difference. High calcium concentrations (30 mM) induced a decrease in the cell number in both hBMSCs and hDFs (Figure 2a).

To investigate whether CaSR was involved in calcium-induced proliferation of hBMSCs, hBMSCs were treated with moderate-high calcium concentrations (3–5 mM) in the presence or absence of NPS2143, a CaSR antagonist, and changes of cell number were examined by the MTT assay (Figure 2b). Comparison of the cell number showed that NPS2143 lowered the cell number in a moderate-high calcium concentration range, indicating that the increased number of hBMSCs in a moderate-high calcium concentration was induced by the CaSR.

Increased cell number in the presence of a moderate-high calcium concentration might have resulted either through increased proliferation or decreased apoptosis. hBMSCs and hDFs exposed to various calcium concentrations for 48 h were subjected to BrdU incorporation or PI-staining to investigate the extent of proliferation or apoptosis, respectively. The proportion of BrdU-positive cells in hBMSCs significantly increased in moderate-high calcium concentrations, whereas no difference was observed in hDFs (Figure 2c). To examine the effect of calcium concentration on cell survival, hBMSCs and hDFs were treated with various calcium concentrations for 48 h and subjected to propidium iodide (PI) staining without plasma membrane permeabilization. The proportions of living cells were determined by PI (-) fraction by FACS analysis (Figure 2d). Although the data indicated that elevated calcium concentration slightly reduced the survival fractions in hDFs, it was not statistically significant. These results indicate that the increased cell number shown in hBMSCs at moderate-high calcium concentration was not due to decreased cell death, but increased cell proliferation.

### 2.2. The Lasting Effect of Moderate-High Calcium Concentration on Cell Proliferation of hBMSCs

**Figure 3:**
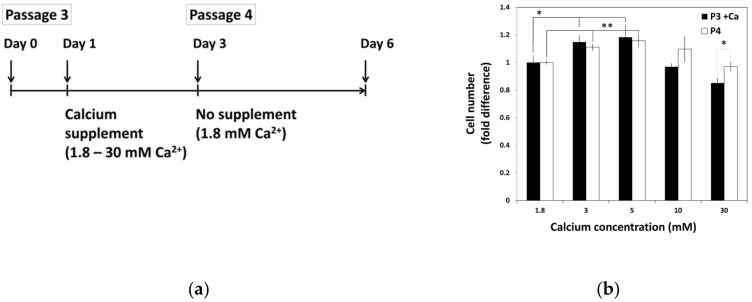
The continuance of hBMSCs proliferation after exposure to moderate-high calcium concentrations. (**a**) The schematic drawing of experiment procedure. hBMSCs were treated with various calcium concentrations for 48 h and sub-cultured to in standard media with normal calcium level. (**b**) Cell growth was evaluated after three days of seeding and compared between passages (P3 + Ca: * 1.8 vs. 3 mM *p* = 0.0293, * 1.8 vs. 5 mM *p* = 0.04481, P4: ** 1.8 vs. 3 mM *p* = 0.0030, ** 1.8 vs. 5 mM *p* = 0.0071).

To examine the effect of calcium withdrawal on proliferation of hBMSCs, hBMSCs were exposed to various calcium concentrations for 48 h before passage. After passage, all groups of cells were maintained in standard media with normal calcium concentration. Three days after the seeding, the extent of cell proliferation was calculated and compared with that of previous passage in various calcium concentrations (Figure 3). This data showed that increased cell growth tendency induced by calcium treatment in moderate-high calcium concentration was maintained after passage, even in the media containing normal calcium concentration.

### 2.3. Influence of Various Calcium Concentrations on Multi-Lineage Differentiation Potentials of hBMSCs

**Figure 4:**
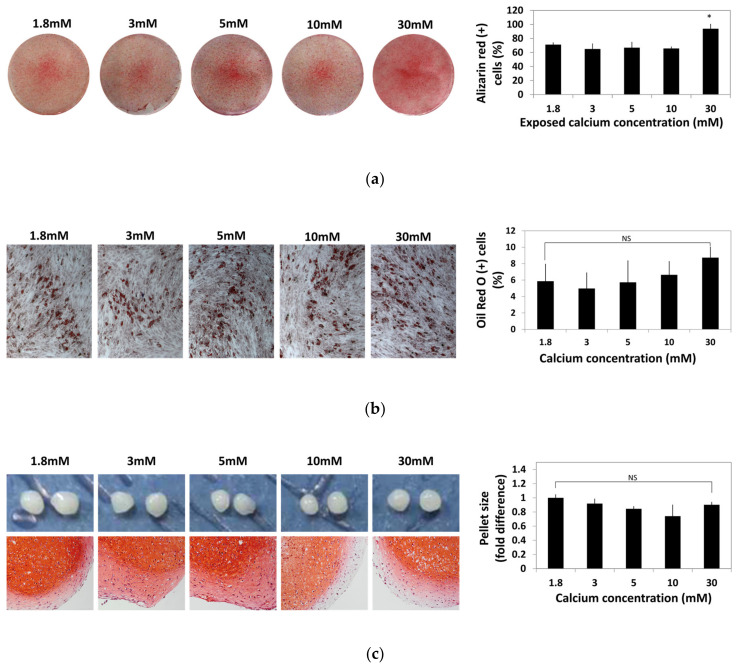
Effect of various calcium concentrations on multi-lineage differentiation potentials of hBMSCs. hBMSCs were treated with various calcium concentrations for 48 h and cultured in differentiation induction media for four weeks. (**a**) Calcium depositions in hBMSCs subjected to osteogenesis were visualized by Alizarin Red S staining. The intensity of Alizarin Red S staining was evaluated by ImageJ software (* *p* = 0.0113). (**b**) hBMSCs subjected to adipogenesis was stained by Oil Red O. The extent of adipogenesis was determined by comparing the proportion of Oil Red O-positive cells normalized by the number of total nuclei. (**c**) After in vitro chondrogenesis, the cartilaginous matrix formation was confirmed by Safranin O staining.

To identify the change caused by the exposure to moderate-high calcium concentration, hBMSCs were exposed to various calcium concentrations for 48 h and cultured in osteogenic, adipogenic, and chondrogenic induction medium for four weeks. hBMSCs differentiated into osteogenic, adipogenic, and chondrogenic lineages were stained with Alizarin Red S, Oil Red O, and Safranin O, respectively. Alizarin Red S-positive cells and Oil red O-positive cells were numerically compared by image analysis using ImageJ software and plotted in a graph (Figure 4).

Osteogenic potential of hBMSCs after exposure to various calcium concentrations did not show significant difference up to a 10 mM calcium concentration. However, the hBMSCs treated with calcium concentrations as high as 30 mM showed increased osteogenic differentiation (Figure 4a). Change in adipogenic potential of hBMSCs after exposure to various calcium concentrations was not significant (Figure 4b). After chondrogenic differentiation, glycosaminoglycans (GAGs) production visualized by Safranin O staining was slightly reduced at the exterior part in the 10–30 mM calcium concentration and the pellet size did not show a significant difference between the calcium concentration groups (Figure 4c). These results show that various calcium concentrations did not impair the multipotency of hBMSCs.

### 2.4. Moderate-High Calcium Concentration Induced Chemotactic Migration of hBMSCs toward Higher Calcium Concentration Gradient through CaSR

**Figure 5:**
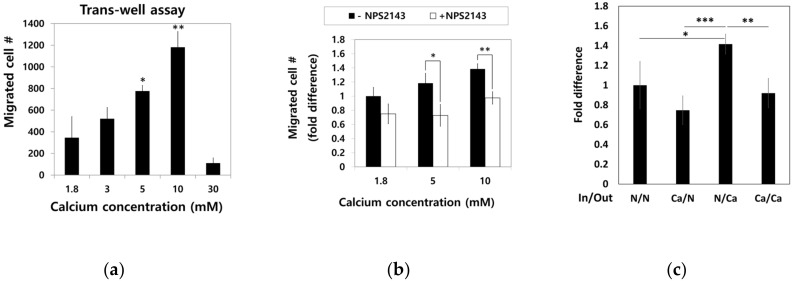

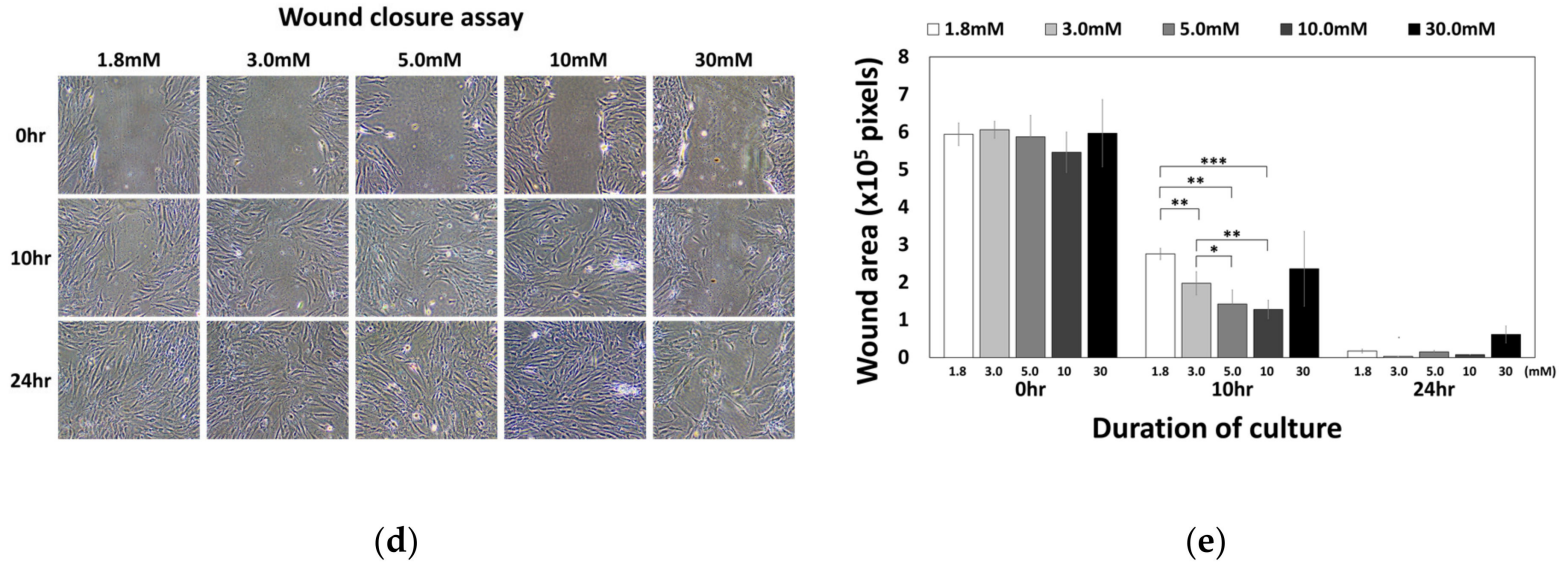
Effect of Ca^2+^_o_ on hBMSC migration behavior. (**a**) hBMSCs in normal calcium media were seeded into millicells across the calcium concentration gradient established by media containing 5–30 mM calcium. After four hours of incubation, the migrated cells were fixed and stained with methyl violet (* *p* = 0.0324, ** *p* = 0.0089). (**b**) To investigate the involvement of CaSR in calcium-induced migration, the migration assay was performed in the presence or absence of 10 μM NPS2143 (* *p* = 0.0317, ** *p* = 0.0085). (**c**) hBMSCs were placed inside the millicell and the media inside and outside of the millicell was controlled as indicated. Ca indicates the media containing 5 mM calcium (* *p* = 0.0242, ** *p* = 0.0015, *** *p* = 0.0003). (**d**,**e**) hBMSCs were subjected to the wound closure assay with various calcium concentrations (1.8 mM vs. 3.0 mM: ** *p* = 0.0094, 1.8 mM vs. 5.0 mM: ** *p*= 0.0013, 1.8 mM vs. 10.0 mM: *** *p* = 0.00007, 3.0 mM vs. 5.0mM: * *p* = 0.0374, 3.0 mM vs. 10.0 mM: ** *p* = 0.0082).

To examine the effect of calcium concentration gradient on the migratory trait of hBMSCs, the cells in standard media were seeded into a millicell insert in 6-well plates and incubated for four hours with outside filled with media containing various calcium concentration. Migratory hBMSCs that passed through the millicell membrane were stained with methyl violet, and only intact cells were counted (Figure 5a). Dose-dependent increase in the migration of hBMSCs was observed up to a 10 mM calcium concentration. However, calcium concentrations as high as 30 mM rather inhibited the migration of hBMSCs.

To investigate whether CaSR was involved in the migration of hBMSCs toward calcium concentration gradient, a migration assay was performed with or without NPS2143 (Figure 5b). While migration of hBMSCs toward the calcium concentration was stimulated by moderate-high calcium concentrations, the presence of the CaSR inhibitor abolished this migratory effect. These results indicate that CaSR is involved in the stimulation of chemotactic migration induced by moderate-high calcium concentration. Although there was no statistical significance, the number of cells that migrated to other side of millicell decreased when cells were seeded with media containing 5 mM Ca^2+^ and the outside was filled with standard media. The migratory behavior of hBMSCs was observed only toward increasing the calcium concentration gradient (Figure 5c), indicating Ca^2+^_o_ exerted their effect by inducing chemotaxis, not chemokinesis. In the wound closure assay, hBMSCs exposed to a moderate-high calcium concentration filled the scratched surface, which is likely due to increased proliferation by a moderate-high calcium concentration (Figure 5d,e).

## 3. Discussion

The CaSR is mainly expressed in tissues involved in calcium metabolism (e.g., parathyroid, kidney, intestine, bone [5,6,7,8]) as well as in cells within the microenvironment where the Ca^2+^_o_ level fluctuates substantially (e.g., osteoclasts and osteoblasts [12,13]). Previous reports have shown that CaSR is expressed in hematopoietic stem cells (HSC) and is involved in HSCs homing from peripheral circulation to bone marrow (BM). In particular, it was suggested that CaSR affects engraftment of HSCs to the endosteal niche in the BM by sensing high Ca^2+^_o_ level near endosteal sites [21,22]. The mechanism affecting HSC homing and engraftment might also affect the homing and engraftment of BMSCs into the BM after mobilization [23]. Several types of cells in osteoblastic lineage express CaSR to promote osteogenic differentiation [24]. Interestingly, when CaSR expression was aberrantly upregulated in entheseal cells, pathological new bone formation was stimulated and resulted in ankylosing spondylitis [25]. Based on the tissue complexity around the enthesis area, the expression of CaSR in entheseal cells might normally be involved in the formation of calcified fibro-cartilage tissue.

Previous studies have shown that high Ca^2+^_o_ stimulates cell proliferation in osteoblasts, myeloma cells, and vascular smooth muscle cells through CaSR-mediated signaling [10,11,12,26,27]. In addition, Ca^2+^_o_-induced chemotaxis of osteoblasts, osteoclasts, and endothelial progenitor cells were mediated by CaSR [12,16,28,29]. The most recent study on the effect of Ca^2+^_o_ on MSC behavior suggested that migration of MSCs toward the Ca^2+^ gradient was induced by OPN, and not by Ca^2+^_o_ itself. However, this work was mostly done with C3H10T1/2, an established cell line from mouse embryonic sarcoma, and Ca^2+^_o_ dependency was checked only with C3H10T1/2 cells [30].

Our results showed that basal level expression of CaSR was higher in hBMSCs compared with hDFs (Figure 1), and moderate-high Ca^2+^_o_ exerted a stimulatory effect on proliferation and chemotactic migration of hBMSCs through CaSR in a dose-dependent manner up to 10 mM (Figure 2 and Figure 5). In our study, the migration assay was confirmed within four hours, which is not enough time for the target gene expressed by CaSR signaling to exert an effect on cell migration.

As a reservoir of calcium and phosphate, bone is undergoing constant remodeling to meet the body’s requirement for calcium and phosphate concentration. During bone remodeling, the action of osteoclasts (OC) and osteoblasts (OB) is in the state of equilibration, resulting in a constant amount of bone mineral content. For this equilibration, several mechanisms exist to couple the action of OC to the activation of OB and vice versa. Cytokines such as IL-1 and IL-6, and growth factors such as TNF-α and TGF-β, RANKL, and OPG are involved in OB–OC coupling [31,32]. Disruption of OB–OC coupling can result in osteopetrosis or osteopenia as exemplified in osteoporosis and Paget’s diseases.

In the BRC, calcium ions are released by OC during bone resorption and the locally elevated calcium level constitutes the concentration gradient [14]. Based on the fact that Ca^2+^_o_ induced chemotactic migration of hBMSCs, it is suggested that hBMSCs, as an osteoblast precursor, migrate toward the BRC in response to the calcium gradient for the initiation of bone formation.

The BRC is sectionalized from bone marrow by the canopy cells, which show similar surface marker expression to osteoblasts. Previous reports have suggested that “OCs have a propensity to position themselves underneath layers of OB-like cells [33,34].” Likewise, BMSCs might position themselves by sensing the chemoattractant’s gradient released by the bone-resorptive action of osteoclasts in the BRC [35,36]. Intact canopy cells can establish the chemoattractant concentration gradient across the BRC and nearby microenvironment to recruit local BMSCs or osteoblast precursors for bone remodeling. Indeed, premature loss or absence of BRC canopies is associated with deficient bone formation in Cushing’s syndrome and post-menopausal osteoporosis [37,38].

**Figure 6:**
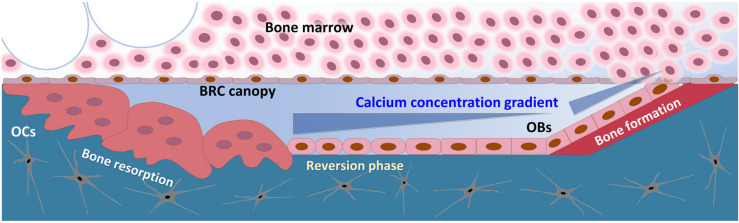
The schematic drawing of BRC (bone remodeling compartment). Compartmentalization of the BRC by canopy cells resulted in the establishment of concentration gradient of the chemoattractant released by the action of bone-resorbing osteoclasts.

In our results, Ca^2+^_o_ concentrations as high as 30 mM even potentiated the osteogenic differentiation potential of hBMSCs (Figure 4a). It is suggested that the calcium concentration gradient around the BRC stimulates proliferation and migration of BMSCs toward the inside of the BRC, and the high calcium concentration (~30 mM) near osteoclasts might induce differentiation of osteoblast precursor cells into osteoblasts, indicating that the calcium gradient established by the BRC may play an important role in bone remodeling by acting as an OB–OC coupling mechanism (Figure 6).

## 4. Materials and Methods

### 4.1. Primary Culture of Bone Marrow Stromal Cells (BMSCs) from Human Bone Marrow Aspirate

hBMSCs were isolated from bone marrow aspirate of patients at Dongguk University International Hospital obtained with informed consent (IRB Approval No. DUIH2012-34). After bone marrow aspirate was diluted with phosphate-buffered saline (PBS), the cells were isolated by density gradient centrifugation using Ficoll-Paque PLUS (GE Healthcare) at 2200 rpm for 20 min. Mononuclear cell fractions were diluted in PBS and centrifuged at 1500 rpm for 5 min at 4 °C. The resultant cell pellets were resuspended in standard medium composed of α-MEM (GIBCO) containing 20% fetal bovine serum (FBS; GIBCO), 10^−8^ M dexamethasone, 0.1 mM ascorbic acid, 1% penicillin/streptomycin, and 2 mM L-glutamine (WelGENE) and seeded at a density of 10^6^ cells per 100 mm tissue culture dish. The cells were incubated at 37 °C in a humidified atmosphere of 5% CO_2_. The medium was changed one day after seeding to wash out the unattached cells. Upon confluence, cells were detached with 0.25% Trypsin/ethylenediaminetetraacetic acid (EDTA) (WelGENE), and passaged at a density of 5 × 10^5^ cells per 100 mm tissue culture dish. The primary cells were used within passage 4.

### 4.2. Primary Culture of Human Dermal Fibroblasts (hDFs) from Human Foreskin Tissue

hDFs were isolated from the foreskin tissue of patients at Chung-Ang University Hospital obtained with informed consent (IBR Approval No. C2009037). Skin tissues were washed in PBS and minced into small pieces. Minced tissues were incubated with 2.4 units/mL dispase overnight at 4 °C. After removal of the epithelial layer, dermal tissues were digested using collagenase type I. Digested dermal tissue was serially filtered through a 70 μm cell strainer and 40 μm cell strainer, and centrifuged at 1500 rpm for 5 min at 4 °C. The cell pellets were resuspended in standard medium and seeded at a density of 10^6^ cells per 100 mm tissue culture dish. The medium was changed one day after seeding. Upon confluence, the cells were detached with 0.25% Trypsin/EDTA (WelGENE), and passaged at a density of 5 × 10^5^ cells per 100 mm tissue culture dish. The primary cells were used within passage 4.

### 4.3. Western Blot Analysis

After the cells were cultured up to confluence, the cells were washed with ice-cold PBS and disrupted by incubating in cell lysis buffer (2 mM Tris-HCl (pH 7.5), 15 mM NaCl, 0.1 mM Na_2_EDTA, 0.1 mM EGTA, 0.1 % Triton, 0.25 mM sodium pyrophosphate, 0.1 mM β-glycerophosphate, 0.1 mM Na_3_VO_4_, and 0.1 μg/mL leupeptin) (Cell Signaling) with the addition of 2 mM PMSF (Sigma-Aldrich, St. Louis, MO, USA) for 30 min on ice. The cell lysates were centrifuged at 12,000 rpm for 20 min at 4 °C and the supernatants were obtained. A total of 40 μg of proteins mixed with sample buffer were loaded and resolved by sodium dodecyl sulfate-polyacrylamide gel electrophoresis (SDS-PAGE), and transferred to a nitrocellulose membrane (Whatman, Maidstone, UK). Membranes were incubated in 5% skim milk in TBS-T for one hour at room temperate and incubated with primary antibody diluted in TBS-T for three hours at room temperature. The membranes were washed with TBS-T and incubated horseradish peroxidase-conjugated secondary antibody (Bio-rad, Hercules, CA, USA) diluted in TBS-T for one hour at room temperature. Specific bindings were detected using Immobilon Western chemiluminescent horse radish peroxidase (HRP) substrate (Millipore, Burlington, MA, USA). The antibodies used for western blot analysis included mouse monoclonal anti-α-tubulin (1:2000; Sigma-Aldrich) and rabbit polyclonal anti-CaSR (1:700; Abcam, Cambridge, UK).

### 4.4. Calcium Chloride and NPS2143 Hydrochloride Treatment

Standard medium contains 1.8 mM calcium chloride (CaCl_2_; Sigma-Aldrich) and this medium was compared as a control. To prepare high Ca^2+^ media, calcium concentrations of standard media were adjusted to 3, 5, 10, and 30 mM with CaCl_2_. Cells were treated with 3, 5, 10, and 30 mM concentration of CaCl_2_ in standard media at passages 3~4. The cells were cultured in high Ca^2+^ media for 48 h at 37 °C in a humidified atmosphere of 5% CO_2_. For inhibitor study, the cells were treated with 10 μM NPS2143 hydrochloride (R&D systems) or absolute ethanol (Merck, Kenilworth, NJ, USA) as a vehicle in the media with various calcium concentrations.

### 4.5. MTT (Thiazolyl Blue Tetrazolium Bromide) Assay

After exposure to various calcium concentrations for 48 h, the cells were incubated in 0.5 mg/mL MTT (Sigma-Aldrich) solution for 4 h at 37 °C. For inhibitor study, the cells were incubated in 0.5 mg/mL MTT solution for 2 h at 37 °C after calcium and NPS2143 treatment. After the removal of medium, the cells were solubilized by dimethyl sulfoxide (DMSO; Sigma-Aldrich), and then the optical density of samples was analyzed using microplate absorbance reader at 550 nm wavelength. The data of the control group were normalized as a value of 1 to calculate relative cell growth.

### 4.6. BrdU (5-Bromo-2′-Deoxy-Uridine) Incorporation Assay

To examine the cellular proliferation rate, the cells were treated with various calcium concentrations for 48 h, and 10 μM BrdU labeling reagent (Roche, Basel, Switzerland) was incorporated for the last 24 h. After fixation, BrdU-incorporated cells were detected using the BrdU Labeling and Detection Kit III (Roche) according to the manufacturer’s protocol. The data were normalized by the optical density of each cell cultured in standard medium.

### 4.7. Propidium Iodide-Incorporated Apoptotic Assay

After the exposure to various calcium concentrations for 48 h, the cells were trypsinized, washed with ice-cold PBS, and resuspended with 1 μg/200 μL propidium iodide (PI; Sigma-Aldrich). PI-positive cells were detected and apoptotic fractions were analyzed using a FACSort flow cytometer (Becton Dickinson, Bergen County, NJ, USA).

### 4.8. In Vitro Multi-Lineage Differentiation Induction

hBMSCs exposed to various calcium concentrations for 48 h were seeded at a cell density of 2 × 10^4^ cells/well in 6-well culture plate (Corning) and maintained in an osteogenic medium composed of α-MEM containing 20% FBS and 1% penicillin/streptomycin, and supplemented with 10^−8^ M dexamethasone, 0.2 mM ascorbic acid, and 10 mM α-glycerophosphate (Sigma-Aldrich). For adipogenic differentiation, hBMSCs exposed to various calcium concentrations for 48 h were seeded at a cell density of 2 × 10^5^ cells/well of 6-well culture plate and maintained in an adipogenic medium composed of high-glucose Dulbecco’s modified Eagle medium (DMEM; Lonza, Basel, Switzerland) containing 10% FBS, 1% penicillin/streptomycin, and supplemented with 10 µg/mL insulin, 10^−7^ M dexamethasone, 0.2 mM indomethacin, and 0.5 mM 3-isobutyl-1-methylxanthine (Sigma-Aldrich). Osteogenic medium and adipogenic medium was changed three times and twice per week, respectively. After the differentiation induction, cultured cells were washed with PBS and fixed with 4% paraformaldehyde (PFA) in PBS. In vitro calcification and lipid droplets were visualized by 2% Alizarin Red S and Oil Red O staining (Sigma-Aldrich). For chondrogenic differentiation, hBMSCs exposed to various calcium concentrations for 48 h were seeded at a cell density of 2.5 × 10^5^ cells per 15 mL conical tube (Corning) and centrifuged at 1500 rpm for 5 min at 4 °C. The pellets were maintained in chondrogenic medium composed of high-glucose DMEM (GIBCO) containing 1% penicillin/streptomycin and supplemented with 10^−7^ M dexamethasone, 0.35 mM proline, 1 × ITS-3, 0.3 mM ascorbic acid (Sigma-Aldrich), and 10 ng/mL TGF-β_3_ (R&D systems). After four weeks of in vitro chondrogenic induction, the pellets were washed with PBS and fixed with 4% PFA. Fixed pellets were paraffin-embedded and sectioned with a microtome. To visualize glycosaminoglycans (GAGs) in cartilaginous matrix, de-paraffinized sections were subjected to Safranin O staining (Sigma-Aldrich).

### 4.9. Migration Assay

hBMSCs cultured in standard medium were seeded into a 12 mm diameter Millicell cell culture insert (Millicell, 12 μm pore, PCF; Millipore, Burlington, MA, USA) at a density of 2 × 10^5^ cells/well, and media containing various calcium concentrations were added in the bottom side of the millicell to set up the calcium gradient. The cells were incubated for four hours at 37 °C and fixed with 4% PFA. After washing with PBS, the cells were stained with saturated methyl violet (Sigma-Aldrich) solution for 20 min and the cells attached to the upper side of the millicell were removed by wiping with cotton swabs. Only intact migrated cells were counted. For inhibitor study, hBMSCs were seeded at a density of 5 × 10^4^ cells/well, and treated with or without 10 μM NPS2143 both in the upper side and bottom side of the millicell. Media containing various calcium concentrations were added in the bottom side of the millicell. Methyl violet-stained migrated cells were lysed by isopropanol and quantified using a microplate absorbance reader at a 550 nm wavelength. These data were normalized by the optical density of cells with added standard medium both in the upper side and bottom side of the millicell.

### 4.10. Statistical Analysis

The results were expressed as the mean ± the standard deviation (SD). The statistical differences between groups were analyzed by the unpaired Student’s *t*-test. *p* value (*p*) < 0.05 was considered to indicate statistical significance.

## Data Availability

The data that support the findings of this study are available from the corresponding author upon reasonable request.

## Supplementary Materials

The following are available online at https://www.mdpi.com/1422-0067/22/1/325/s1.

## Abbreviations

BM	Bone marrow
BRC	Bone remodeling compartment
Ca^2+^_i_	Intracellular calcium
Ca^2+^_o_	Extracellular calcium
CaSR	Calcium-sensing receptor
GAGs	Glycosaminoglycans
hBMSCs	Human bone marrow stromal cells
hDFs	Human dermal fibroblasts
HSCs	Hematopoietic stem cells
IL	Interleukin
OB	Osteoblast
OC	Osteoclast
OPG	Osteoprotegerin
OPN	Osteopontin
PFA	Paraformadehyde
PI	Propidium iodide
RANKL	Receptor activator of nuclear factor kB ligand
TGF-β	Transforming growth factor-beta
TNF-α	Tumor necrosis factor-alpha
