# Supplementary material for: CaSR-Mediated hBMSCs Activity Modulation: Additional Coupling Mechanism in Bone Remodeling Compartment
Source: Int J Mol Sci. 2020 Dec 30;22(1):325. doi: 10.3390/ijms22010325 (PMC7795180; doi:10.3390/ijms22010325)
Supplement: Supplementary file 1 [file ijms-22-00325-s001.pdf]

Supplement 1

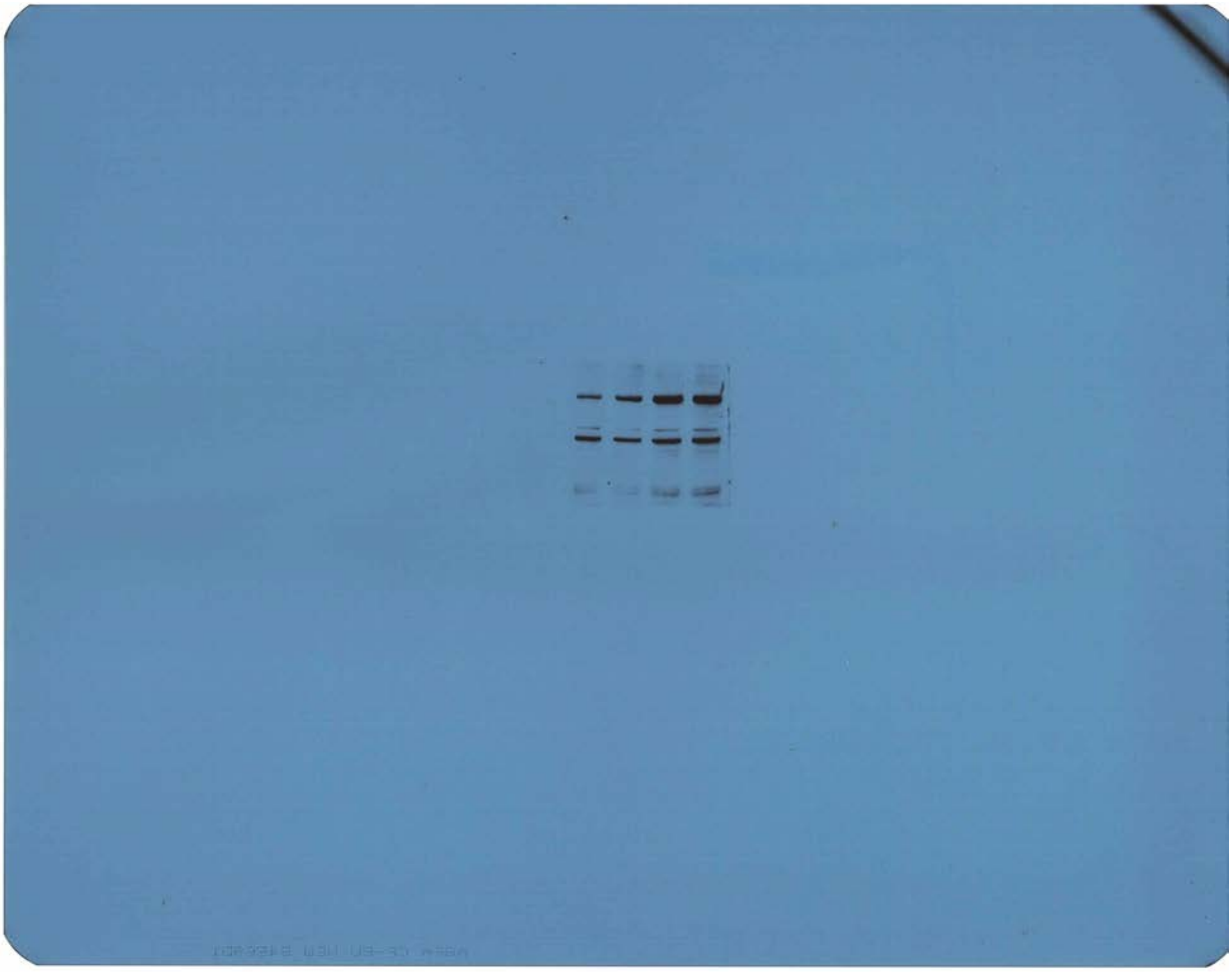

WB: Calcium-sensing receptor (CaSR)
Lane 1: hDF
Lane 2: hDF
Lane 3: hBMSC
Lane 4: hBMSC

Supplement 2

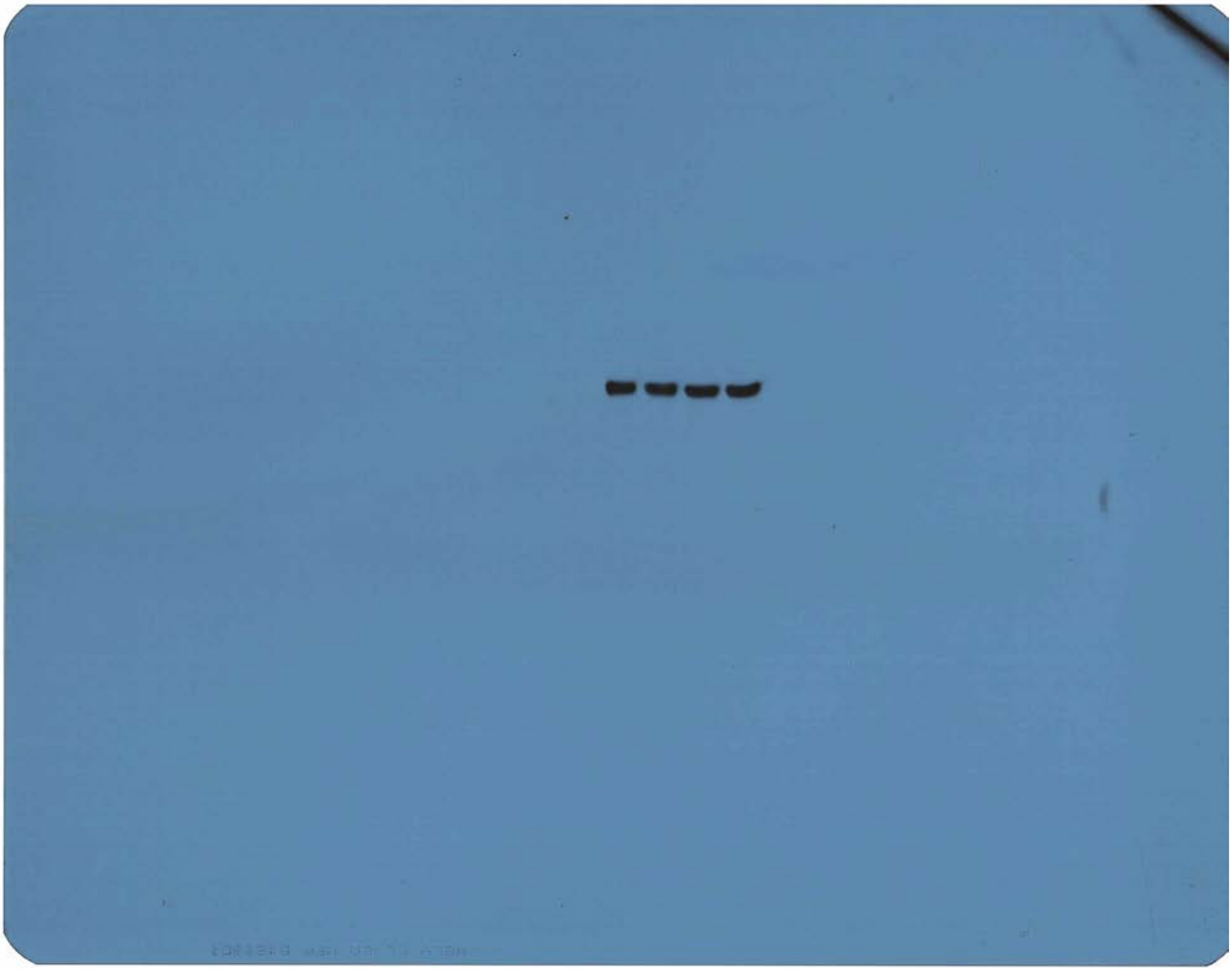

WB: α-tubulin
Lane 1: hDF
Lane 2: hDF
Lane 3: hBMSC
Lane 4: hBMSC
